# Donor milk intake and infant growth in a South African neonatal unit: a cohort study

**DOI:** 10.1186/s13006-018-0183-8

**Published:** 2018-09-04

**Authors:** Hayley Sparks, Lucy Linley, Jennifer L. Beaumont, Daniel T. Robinson

**Affiliations:** 1 Northwestern University Feinberg School of Medicine, Chicago, IL USA; 2 Division of Neonatology, School of Child and Adolescent Health, University of Cape Town and Mowbray Maternity Hospital, Cape Town, South Africa; 3 Terasaki Research Institute, Los Angeles, CA USA; 4 Department of Medical Social Sciences, Northwestern University Feinberg School of Medicine, Chicago, IL USA; 5 Department of Pediatrics, Northwestern University Feinberg School of Medicine, Chicago, IL USA; 6 Ann & Robert H. Lurie Children’s Hospital of Chicago, Chicago, IL USA

**Keywords:** Milk bank, Human milk, Donor milk, Infant growth, Very low birth weight, Premature infant

## Abstract

**Background:**

Implications of donor milk feedings on infant growth in resource limited settings remain uncertain. This knowledge gap includes the impact of donor milk availability on infant intake of mother’s own milk. Therefore, this investigation aimed to measure intake and growth in infants receiving donor milk when born to women from resource limited backgrounds with high rates of human immunodeficiency virus (HIV).

**Methods:**

A retrospective cohort study enrolled eligible infants admitted to a South African combined neonatal intensive and secondary high care unit, within a one year admission period during 2015, with signed consent for donor milk feedings. A certified milk bank provided donor milk. Daily nutritional intake during the first month was recorded. Details included proportional intake of donor milk, mother’s own milk and infant formula. The primary outcome of infant growth velocity from day back to birth weight to discharge was calculated when length of stay was ≥14 days. Analyses primarily used T-tests; mixed effects models compared weekly calorie intake.

**Results:**

One hundred five infants with donor milk consent were born at 30.9 ± 3.6 weeks of gestation, weighing 1389 ± 708 g. Forty percent of mothers had HIV. Infant growth velocity did not differ based on percent of feedings as donor milk (≥ 50%: 11.8 ± 4.9 g/kg/d; < 50%: 13.5 ± 5.3 g/kg/d; *p* = 0.3). Percent of feedings from donor milk was similar based on maternal HIV status (positive: 31 ± 25%; negative: 36 ± 29%; *p* = 0.4), as was percent of feedings as mother’s milk (positive: 53 ± 35%; negative: 58 ± 30%; *p* = 0.4). Calorie intake increased markedly during the first two weeks and then plateaued (*p* < 0.0001).

**Conclusions:**

Donor milk feedings in higher proportions did not further impair growth of infants managed in a South African combined neonatal intensive and secondary high care unit with growth rates already below reference ranges. The provision of donor milk contributed to feedings being composed of primarily human milk during the first month. Increasing early calorie intake may improve infant growth in this center.

## Background

Pasteurized donor milk (DM) availability in South African hospitals has increased substantially during the last twenty years with the establishment of the Human Milk Banking Association of South Africa (HMBASA) [1]. Low-cost technology supports pasteurization in resource limited settings [2]. Increased access to DM through these milk banks coincides with recommendations that DM be provided to preterm and low birth weight infants when a mother’s own milk (MOM) is not available or sufficient for feedings [3, 4]. Donor milk availability can allow feedings of critically ill infants born to women in resource limited settings and with high rates of human immunodeficiency virus (HIV) to be predominantly composed of human milk [5]. Its use should be expected to occur simultaneously with lactation support given a priority for providing MOM [6, 7].

Using DM in place of formula in conjunction with mother’s milk reduced the risk of prematurity associated morbidities including necrotizing enterocolitis (NEC) and acute and chronic respiratory disorders, although not in all situations [8–12]. Heating milk alters its composition. Loss of bile salt-stimulated lipase activity, which occurs during any heating method, reduces infant fat absorption [13]. Trace elements, growth factors and cytokines are preserved during pasteurization [14, 15]. However, bioactive hormones including insulin and adiponectin are reduced [16], as may be oligosaccharides depending on the heating method [17]. The cumulative impact of these alterations on infant health, including growth patterns, remains unclear.

The relative proportions of DM versus MOM used for feedings influence clinical outcomes resulting from DM exposure [18]. Insufficient research has evaluated the impact of DM feedings on the outcomes of hospitalized infants in resource limited settings with high rates of maternal HIV. Identifying any impact on infant growth and implications of DM availability on volumes of MOM fed to high-risk infants would establish important foundations upon which nutrition provided to these infants may be improved. Thus, we aimed to measure growth in high-risk infants exposed to DM and the associated nutritional intake patterns when cared for in a sub-Saharan secondary care center managing women from a resource limited background with a high burden of HIV.

## Methods

### Study design

A single center, retrospective study measured infant growth as related to DM exposure during a one-year admission period within which no significant changes in standards of clinical care occurred. As a secondary care center, Mowbray Maternity Hospital (MMH) in Cape Town, South Africa serves disadvantaged women from the immediate referral area surrounding the hospital. In addition, MMH serves women referred from level one facilities including Midwife Obstetric Units, which are midwife-run primary public care centers for those unable to buy private insurance. The neonatal unit (NU) contains 73 beds, six of which are tertiary care beds and the rest designated as secondary care. One attending neonatologist and two attending pediatricians perform clinical duties daily.

Medical records of infants admitted between January 1, 2015 and December 31, 2015 were screened for eligibility. Eligibility was defined as having a signed consent form for DM feedings. All eligible infants had a copy of this signed consent form permitting use of DM feedings located in both the medical record and a separate satellite maintained by the DM bank, Milk Matters. Exclusion criteria included a major congenital anomaly or aneuploidy.

### Donor milk and nutrition management

The MMH NU standards for nutrition management have remained consistent prior to, during and since the study period. A priority in the MMH NU is to prescribe human milk feedings, either MOM or DM. Feeds are initiated on Day 0–1, with colostrum and trophic feeds as available. Feeding initiation volumes range 60–100 ml/kg/d with daily advancements of 20–30 ml/kg/d to a maximum of 160 ml/kg/d in term infants and 200 ml/kg/d in preterm infants. Feedings are held or the rate of advancement lowered for signs of gastrointestinal distress, including emesis and abdominal distension. Preterm infant feedings of expressed milk have human milk fortifier added after infants achieve full feeding volumes. For those infants not tolerating feeds by 48–72 h of age, parenteral nutrition (PN) may be initiated, although uncommonly given insufficient resources for safety monitoring of intravenous infusions.

Milk Matters, a member of HMBASA, provides DM to the MMH NU. Milk Matters uses the Holder pasteurization method involving heating milk to 62.5 °C for 30 min and cooling to 4 °C over 60 min for a total pasteurization cycle time of 120 min. Any mother’s milk expressed at the hospital and stored for future use also undergoes Holder pasteurization, regardless of HIV status. Mothers may also express milk for immediate use or breastfeed the infant at the bedside. If a mother is HIV positive and expresses a small amount of milk at the bedside for immediate use, the milk undergoes flash pasteurization prior to use for feeding. Flash pasteurized milk is rapidly boiled in a glass jar within a water bath. For infants able to feed by mouth and born to HIV negative women, expressed milk may be provided via cup feeding or infants may breastfeed.

Eligibility for DM feedings requires fulfillment of at least one infant and one maternal criterion. Infant criteria include: 1) prematurity (birth weight < 1500 g), 2) recommencement of feedings after surgery or ‘abdominal mischief,’ or 3) preterm or ill infants born to HIV positive women. Maternal criteria for mothers of qualifying infants as detailed above are 1) insufficient supply of expressed human milk due to breast surgery or illness 2) insufficient supply during 1st-3rd day postpartum in spite of ongoing efforts at milk expression, or 3) a contraindication to using own milk (chemotherapy, serious maternal substance abuse). When criteria are met, parents are informed of the option for DM and then written, informed consent is obtained prior to implementing DM feedings. Although the hospital purchases DM, families are not informed of the cost or asked to pay for DM. Appropriateness of DM feedings is reevaluated every two weeks and a new consent form signed indicating the continued need and fulfillment of criteria. When infants no longer fulfill criteria, MMH transitions infants to preterm formula if needed. Milk Matters stores copies of the consents for DM at a satellite location for at least 10 years.

### Outcomes

The primary outcome was infant growth velocity (GV) from day back to birth weight through discharge, calculated using the exponential model [19]. Routine anthropometric monitoring in the MMH NU included recording daily weight and weekly head circumference. Infant lengths were not routinely recorded due to concerns about accuracy of the measurement and high patient volume. Standard deviation (Z) scores, gender specific, were calculated based on gestational age at birth and postmenstrual age at discharge using the Fenton growth curves for reference [20].

The recorded details of infant feedings included: indications for prescribing DM, types of feedings prescribed throughout hospitalization, and daily feeding intake (actual amounts received, not prescribed) for the first 28 days or through discharge if earlier. Additional characteristics abstracted from medical records included maternal demographics and infant descriptors: maternal age and HIV status, disorders of pregnancy, mode of delivery, infant inborn status, prevalence of infant morbidities acquired during hospitalization (e.g. sepsis, respiratory disease, abdominal mischief/NEC), infant length of stay (LOS) and mortality.

### Statistical analysis

Infant GV between the day back to birth weight and discharge was calculated for infants whose LOS lasted ≥14 days. Subgroup analyses were performed for very low birth weight (VLBW) infants and by whether infants were born prior to 32 weeks of gestation. Mixed effects models compared infant weekly volume and energy intake. Energy content of unfortified human milk was assumed to be 67 kcal/dL [21]. Standard summary statistics were calculated for all variables as appropriate and all analyses were performed using SAS 9.4. Two-sample t-tests were used to compare growth and nutrition measures between groups.

## Results

Donor milk consent existed for one hundred five infants during the study period (Table 1). Amongst mothers of these infants (*n* = 99; 27.9 ± 6.3 years old), 40 % were HIV positive (Table 1). Infant birth weight differed by maternal HIV status (HIV positive: 1790 ± 859 g; HIV negative: 1109 ± 390 g; *p* < 0.001). The most common infant indication for prescribing DM was prematurity (Table 2). Noteworthy infant outcomes included low rates of NEC and infrequent use of PN (Table 1).

**Table 1:** Demographic characteristics of mothers and infants

Maternal characteristics (*N* = 99)	*N* (%)^a^
Antenatal care	65 (73)
Primigravid	19 (21)
HIV Infection	40 (40)
Active tuberculosis	3 (3)
Gestational proteinuric hypertension	17 (19)
Gestational diabetes	0
Substance abuse during pregnancy	32 (35)
Multiple gestation pregnancy	18 (20)
Antenatal steroids	38 (41)
Chorioamnionitis	10 (11)
Cesarean delivery	43 (48)
Infant characteristics (*N* = 105)	Mean ± SD or *N* (%)
Gestational age, weeks	30.9 ± 3.6
Birth weight, grams	1389 ± 708
Inborn^b^	68 (69)
Male gender	49 (50)
Respiratory distress syndrome	43 (44)
Patent ductus arteriosus	17 (18)
Surfactant administration	29 (30)
Intraventricular hemorrhage	23 (27)
Infection	21 (22)
Infant characteristics	N (%)
Necrotizing enterocolitis	3 (3)
Any total parenteral nutrition use	4 (5)
Mortality during admission	10 (10)

^a^Missing values range *n* = 7–10 for maternal characteristics, excluding HIV infection, and *n* = 7–9 for infant characteristics aside from intraventricular hemorrhage (*n* = 21 missing) and parenteral nutrition use (*n* = 20 missing); percentages reported reflect available information

^b^Refers to infants born at Mowbray Maternity Hospital, as opposed to infants born elsewhere and then transferred to the neonatal unit for ongoing care

**Table 2:** Indications for prescribing donor milk to infants (*N* = 105)

Infant Criteria	*N* (%)
Preterm infant (< 1500 g)	75 (71)
Recommencement of feeds for babies after surgery or with ‘abdominal mischief’	11 (10)
Preterm or ill babies of HIV infected mothers	31 (30)
Maternal Criteria for Infant Eligibility	
Insufficient supply of mother’s own milk due to breast surgery or illness	42 (40)
Building up own supply 1st-3rd days post partum when pasteurizing	34 (32)
Transportation or financial difficulties and no rooming is available	19 (18)
Contraindications to using own milk (chemotherapy, serious maternal substance abuse)	1 (1)

Note: More than one indication may apply

### Infant growth

Seventy six infants were hospitalized ≥14 days and 64 had day back to birth weight accurately recorded in the medical record. For those infants, GV through discharge was 13.1 ± 5.2 g/kg/d; this rate was similar for infants whether they were born prior to the 32nd week or later (*p* = 0.99). Weight Z scores decreased from birth to discharge (ΔZ: − 1.46 ± 0.76) as did head circumference Z scores (− 0.47 ± 0.98). Most of these infants were VLBW (*n* = 56) and their GV was 13.8 ± 4.4 g/kg/d. Growth velocity was similar whether infants received more or less than 50% of their feeds as DM during the first month (Table 3). Infant GV did not differ by maternal HIV status (Table 3).

**Table 3:** Growth of discharged infants with admission ≥14 days, values reported as mean ± sd

Growth Velocity by Donor Milk Consumption	< 50% of feeds as DM	≥ 50% of feeds as DM	*p*
All discharged infants	*n* = 48	*n* = 16	0.3
	13.5 ± 5.3	11.8 ± 4.9	
Discharged very low birth weight infants	*n* = 41	*n* = 15	0.2
	14.3 ± 4.7	12.7 ± 3.4	
Growth Velocity by Maternal HIV Status	No HIV Infection	HIV Infection	
All discharged infants	*n* = 42	*n* = 22	0.6
	13.4 ± 4.3	12.6 ± 6.7	
Discharged very low birth weight infants	*n* = 42	*n* = 14	0.1
	13.4 ± 4.3	15.3 ± 4.6	

### Infant nutrition

Amongst infants discharged from the NU, 95% received MOM, 95% received DM and 50% received preterm infant formula at any time during hospitalization. For infants receiving < 50% of feeds as DM, the proportion of feedings as DM was 21 ± 15% in the first month. Conversely, for those receiving predominantly DM, the proportion of feedings as DM was 74 ± 16%. The median percentage of feedings as any human milk during the first month for discharged infants was high (100, IQR 93,100) and proportions of feedings as MOM versus DM did not appear to be impacted by maternal HIV status (Table 4). Only five infants were fed MOM exclusively throughout hospitalization. Eighty five percent of discharged infants received human milk fortifier for some feedings. For infants with a LOS ≥ 14 days, feeding volumes and calorie intake showed significant increases during the first two weeks and then consistent intake during the remainder of the first month, and gestational age did not appear to modify this association when comparing infants born prior to the 32nd week or later (*p* > 0.1) (Table 5). For VLBW infants hospitalized for over two weeks (*n* = 63), mean calorie intake during the first month was 111 ± 17 kcal/kg/d.

**Table 4:** Distribution of Milk Intake by Maternal HIV Status, values reported as Mean ± SD

	No HIV Infection	HIV Infection	*p*
All discharged infants^a^	*n* = 52	*n* = 34	
Percentage of feedings as mother’s own milk during first 28 days	58 ± 30	53 ± 35	0.4
Percentage of feedings as donor milk during first 28 days	36 ± 29	30 ± 25	0.4
Discharged very low birth weight infants	*n* = 48	*n* = 15	
Percentage of feedings as mother’s own milk during first 28 days	58 ± 30	67 ± 31	0.3
Percentage of feedings as donor milk during first 28 days	37 ± 30	28 ± 27	0.3

^a^Missing data for *n* = 9

**Table 5:** Weekly intake for discharged infants with an admission duration ≥14 days, values reported as mean ± sd

	Week 1	Week 2	Week 3	Week 4	*p*
Gestational age, < 32 weeks	*n* = 39	*n* = 45	*n* = 45	*n* = 44	
Fluid Intake, ml/kg/day	70 ± 29	172 ± 23	168 ± 23	169 ± 15	< 0.0001
Energy Intake, kcal/kg/day	47 ± 20	128 ± 20	129 ± 20	131 ± 14	< 0.0001
Gestational age, ≥ 32 weeks	*n* = 25	*n* = 25	*n* = 24	*n* = 18	
Fluid Intake, ml/kg/day	66 ± 32	158 ± 32	165 ± 37	160 ± 27	< 0.0001
Energy Intake, kcal/kg/day	45 ± 22	117 ± 25	125 ± 31	118 ± 25	< 0.0001

## Discussion

While concern remains that DM feedings negatively impact growth for human milk-fed infants, this analysis suggests that increasing amounts of DM to > 50% of feedings in the first month does not slow infant growth. However, it is noteworthy that the GV of all infants in this South African cohort falls below fetal reference ranges [22]. For those infants with a signed consent in the MMH NU, DM availability facilitated high rates of feedings as human milk during the first 28 days. We detected no difference in GV related to maternal HIV status. We believe the difference in infant characteristics, including birth weight, based on maternal HIV status to be a consequence of infant inclusion criteria for DM at MMH. Infants of mothers with HIV may receive DM while an infant with a comparable degree of illness, born to a mother without HIV, might not qualify. Specifically, an ill infant born to a mother with HIV could receive DM while DM would not be used for the ill infant born to a mother without HIV if sufficient MOM were available. In a population of women with high rates of HIV infection, DM availability did not appear to decrease amounts of MOM fed to their infants. Availability of DM has previously improved rates of breastfeeding at discharge [9].

Implementation and acceptance of DM feedings in communities with high rates of maternal HIV and other clinical settings appears feasible [23]. Still, clinician sensitivity to women’s concerns towards use of DM feedings is important. Parents relay concerns about disease transmission through milk, including HIV, yet increasing parental familiarity with screening and pasteurization processes may reduce such concern [23]. Independent of safety concerns, using DM creates stress for many mothers resulting from the need to rely on milk expressed from other women [24]. Without surprise, affording parents sufficient time and opportunities to discuss DM with clinicians helps clarify misunderstandings and may ease concerns. As families continue to consider DM as a viable option for feedings, detailing growth and outcomes for infants using DM in sub-Saharan Africa is also necessary for improving infant care.

Donor milk feedings should not be singled out as the sole contributor to the measured GV of these infants. Prompt initiation, advancement and fortification of feedings contributed to a weekly caloric intake that ultimately exceeded recommendations [21], yet not until after the first week. Also, as VLBW infants made up a considerable proportion of this cohort, average caloric intake suggests that infants with larger birth weights achieve higher caloric intake than smaller infants. Thus, early caloric intake appears to be a modifiable factor in efforts aimed at improving growth. Early PN is a commonly used method for minimizing early caloric deficits [25]. However, resources and personnel required to maintain safe intravenous infusions currently precludes the use of PN in many infants. Protein concentrations in DM, even when combined with human milk fortifiers, may not meet estimated requirements [21, 26]. Also, in consideration of the standards for processing human milk used in the MMH NU, much of the milk used (any DM, all stored MOM regardless of a mother’s HIV status and all expressed milk from HIV positive mothers) undergoes some method of heating prior to infant feeding. In addition, the preterm infant’s endogenous lipase capabilities are limited [27]. Cumulatively, these combined factors would be expected to exacerbate fat malabsorption as opposed to other clinical scenarios where MOM is not heated. Access to a safe adjuvant to facilitate fat absorption would be expected to mitigate the poor growth observed in comparable populations, however, a well-tolerated option is not currently available [28].

Delayed availability of MOM was the most common maternal indication for DM feedings. This is consistent with other reports [1]. Delayed onset of lactation after preterm delivery is not uncommon [29]. In this context, the establishment of DM availability for preterm infants during insufficient supply of MOM is consistent with global public health recommendations [3, 4]. Indicators such as rates of breastfeeding at time of discharge from neonatal units suggest DM availability may improve use of MOM [9, 30], but in some scenarios it appears to hinder intake of MOM [30]. It is possible that discussions of DM with parents emphasize the importance of human milk for feedings, and when combined with lactation support, increased intake of MOM should be expected in some settings [31]. Thus, ongoing lactation support is critical for women who deliver critically ill, preterm infants and for women who may be temporarily separated due to infant transfer to another facility in the immediate postnatal period [32].

In this cohort of infants with consent to use DM, a small but meaningful proportion (5%) of infants ultimately never used DM and received their mother’s milk exclusively. This scenario of anticipated need for supplemental milk without eventual need has been documented in the setting of large, multicenter randomized controlled trials. In a Dutch trial comparing DM versus formula to fill the gap when needed for feedings, 3.2% of randomized infants received all feedings as MOM [33]. In a separate Canadian trial comparing DM and formula, approximately 28% of infants ended up feeding only their mother’s milk [34]. These subsets of infants and their mothers appear worthy of attention in efforts to define clinical scenarios in which a clinician may predict the need for supplementation and yet ultimately there was no need.

Approximately half of infants received formula at some point during hospitalization. Feeding composition during the first month suggests that formula use occurred later in hospitalization when needed. High rates of human milk feedings and later exposure to formula may in part explain the low rates of NEC in this population [8]. In comparison, overall rates of NEC for VLBW infants cared for in the MMH NU are 1.6%.

The long-term implications of the measured growth outcomes remain to be determined, although infant growth rates act as surrogate markers of whether brain development is optimal. Greater weight gain and head growth during initial hospitalization were previously associated with higher developmental testing scores in preterm infants [35]. Although length data were not available for these infants, linear growth during hospitalization as well as post discharge is a valid predictor of neurodevelopmental impairment in preterm infants [36].

Inherent limitations must be acknowledged related to the retrospective study design, including missing data. Also, despite considerable detail of nutrition and fluid intake during the first 28 days, daily intake throughout hospitalization may have revealed additional nutritional factors that contributed to the observed growth patterns. Additionally, growth was only measured during the hospital stay. Post discharge outcomes are necessary to draw robust conclusions about any lasting implications of early nutrition. This investigation was unable to identify infants who may have qualified for DM feedings but parental preference was to not provide consent. Similarly, we could not ascertain reasons why parents may have preferred not to consent for DM in those scenarios. However, clinicians in the MMH NU observe this to be an uncommon occurrence. Regardless, a prospective investigation examining the percentage of guardians choosing not to consent for DM, their reasoning why, and how these infants grow relative to those who do have signed consents would be important for a comprehensive understanding of the implications of DM feedings.

## Conclusion

In conclusion, the use of DM for feeding infants managed in a secondary South African Neonatal Unit was associated with suboptimal infant growth. As intended, DM availability allows delayed exposure to formula and can contribute to maintaining high proportions of human milk feedings for high-risk infants. The consideration of all circumstances surrounding the nutritional management in any single center appears vital to understanding how to optimize infant outcomes associated with DM feedings. Lactation support, relative amounts of a mother’s milk availability and how MOM is handled prior to feedings likely create unique and individual circumstances which require attention during clinical and research efforts aimed at improving feeding quality and infant outcomes.

## Abbreviations

DM: Donor milk
GV: Growth velocity
HIV: Human immunodeficiency virus
LOS: Length of stay
MMH: Mowbray Maternity Hospital
MOM: Mother’s own milk
NEC: Necrotizing enterocolitis
NU: Neonatal unit
PN: Parenteral nutrition
VLBW: Very low birth weight
